# Androgens modulate endometrial function

**DOI:** 10.1007/s00795-025-00430-6

**Published:** 2025-03-10

**Authors:** Ko Yamagata, Yousuke Mizuno, Yumi Mizuno, Shunnsuke Tamaru, Takeshi Kajihara

**Affiliations:** 1 Department of Obstetrics and Gynecology, Saitama Medical University, 38 Morohongo, Moroyama, Iruma-gun, Saitama, Japan; 2 Division of Morphological Science, Biomedical Research Center, Saitama Medical University, Saitama, Japan; 3 Division of Experimental Animal, Biomedical Research Center, Saitama Medical University, Saitama, Japan

**Keywords:** Androgens, Endometrium, Decidualization, Implantation, Endometriosis

## Abstract

Human endometrium is the major target tissue for sex steroid hormones. The circulating steroid hormones in normal ovulatory cycles accurately control the proliferation and differentiation of the endometrial cells. Androgens, such as testosterone and 5α-dihydrotestosterone, are a type of sex steroid hormones that mainly function in the differentiation, development, and maintenance of male sexual characteristics. Although androgens are best known for their role in male reproduction, the androgen receptor is present in both male and female reproductive organs and is essential for normal reproductive function. Recently, a series of evidence suggests that androgens contribute to endometrial physiology and pathologies. However, the roles of androgens in the endometrium remain insufficiently understood, with contradictory findings being reported. This review summarizes the studies that show the role of androgens in regulating the physiological conditions of the endometrium and the implantation process, and endometrial pathology including endometriosis and others.

## Introduction

The human endometrium is the major target tissue for sex steroid hormones, including estrogen and progesterone. In normal ovulatory cycles, the proliferation and differentiation of the endometrial cells are accurately controlled by the circulating steroid hormones. Notably, the progesterone level increase in the postovulatory phase induces profound remodeling of the estrogen-primed endometrium, characterized by significant growth and coiling of the spiral arteries, secretory transformation of the glands, and decidualization of the stromal compartment. Successful implantation depends on the interaction between a well-developed embryo and a “receptive” endometrium. The duration of endometrial receptivity in the cycle is limited, designated as the “implantation window,” wherein the endometrium allows blastocyst implantation. The endometrium becomes receptive approximately 6 days after ovulation and remains receptive for up to 2–4 days [1–4].

Although androgens are best known for their role in male reproduction, the androgen receptor (AR) is also present in female reproductive organs and is essential for normal reproductive function [5, 6]. Furthermore, androgens have been reported to contribute to endometrial physiology and pathologies such as endometriosis and endometrial cancer (EC). However, relative to the role of estrogen and progesterone on physiological pathological endometrium, information on the roles of androgens in the endometrium is still limited, with contradictory findings being reported. In this review, we summarize the studies that describe the role of androgens in regulating the physiological conditions of the endometrium and the implantation process and focus on the association between androgens and endometrial pathology, including endometriosis and EC.

## Search strategy and selection criteria

We conducted a narrative analysis in the literature review. The PubMed and Google Scholar were searched for literature published up to March 31, 2024, combining the following keywords: “Androgen,” “Endometrium,” “Implantation,” “Decidualization,” “Endometriosis,” and “Endometrial Cancer.”

## Characters and roles of androgen

Androgens, which include testosterone and 5α-dihydrotestosterone (DHT), are a type of sex steroid hormones that mainly participate in the differentiation, development, and maintenance of male sexual characteristics. Androgen has a four-ring structure with C17 carbon, indicating a typical steroid skeleton, which is important in the biological activity as a hormone; it also has ketone and hydroxyl groups. Androgen is mainly produced in the testis; adrenal glands and ovary in females also produce it. Initially, cholesterol is transferred to pregnenolone in the mitochondria by the cholesterol side-chain cleavage enzyme P450scc (CYP11A1) [7]. Then, pregnenolone is hydroxylated to 17α-hydroxypregnenolone by the enzyme 17α-hydroxylase (P450c17) in the adrenal cortex [7]. 17α-hydroxypregnenolone is then converted to dehydroepiandrosterone (DHEA) by the 17,20-lyase activity of P450c17 [7]. DHEA is further converted to androstenedione by 3βHSD, and finally androgens such as testosterone are produced by enzyme AKR1C3 [7]. There are also various mechanisms to degrade or convert androgen to the other metabolites. Those mechanisms are important for maintaining androgens as appropriate levels, and have crucial roles in various physiological processes, such as reproductive health in both male and female, influencing sexual behavior, bone density, and fat distribution, and so on. Testosterone is converted to dihydrotestosterone (DHT) by the enzyme 5α-reductase [8]. Aromatase, encoded by the Cyp19a1 gene, catalyzes the conversion of testosterone to estradiol in various tissues, including the brain and vascular endothelium in mice, which is essential for regulating male sexual behavior, and have crucial process in male vascular endothelium [9, 10].

Androgen is widely distributed throughout the body, affecting the entire body. Its function is directly related to male sexual formation, maintenance of male secondary sexual characteristics, development of sexual desire, and sperm development and fertility [11]. The androgen signaling is also involved in various physiological pathways beyond male sex differentiation, such as muscle formation, body hair formation, and vocal cord development, through various tissues and organs. Moreover, it influences red blood cell production through erythropoietin stimulation, and blood lipid amount. Androgen is also produced in females as well [12], performing several functions, such as muscle and bone density maintenance and sexual desire improvement. Moreover, androgen signaling is related to the pathogenesis of several cancer types, especially prostate cancer [13, 14]. It stimulates prostate cancer cells to proliferate, resulting in rapid tumor growth. It also inhibits apoptosis, allowing the tumor to survive [13, 14]. In addition, some breast and ovary cancer types are promoted by androgen signaling via tumor growth progression and apoptosis inhibition. Several approaches are attempted to prevent and treat cancers by inhibiting androgen signaling. As described above, androgen is related to various physiological and pathological processes in the body.

## AR and the signaling pathways

AR is a nuclear receptor protein. Androgen specifically binds to AR. Once activated, the AR transfers the stimulation of androgens in cells [15]. AR is mainly expressed in the cells of male reproductive organs, including the testes and prostate, but it is also found in other tissues, such as the skeletal muscle, skin, adrenal ground, and ovary in females, transmitting various stimuli within the body. In cells, AR normally exists in cytosols as a monomer. When bound with androgen, AR is activated, subsequently changing to a dimer [16], which subsequently moves into the nucleus. Androgen-bound AR dimer in the nucleus is then bound to specific DNA sequences [androgen response elements (AREs)]; thereafter, the expression of specific genes is activated or inhibited. The target genes whose expression is regulated by androgen signaling can be identified mostly by the chromatin-immune precipitation method [17–19]. Of them, prostate-specific antigen (*PSA*) is a major target gene of androgen signaling. *PSA* is mainly expressed in the prostate and is involved in the prostate structure and function by secretion as a part of seminal fluid. In particular, it helps enhance sperm motility and liquefy the seminal fluid to increase the possibility of fertilization [20]. Insulin-like growth factor 1 (*IGF-1*), erythropoietin (*EPO*), and succinate dehydrogenase are also the target genes of androgen signaling. Thus, by controlling the expression of multiple target genes, androgen signaling can be involved in several physiological and pathological pathways in living cells, affecting many individual body regulations.

## Androgens regulate the menstrual cycle in the endometrium and endometrial decidualization

The endometrium is a major target tissue of sex steroid hormones, including estrogen and progesterone. Androgens play an important role in male reproduction. However, both male and female reproductive organs express AR, which is essential for both normal reproductive functions [5, 6]. Although both the epithelial and stromal compartments of the endometrium express the estrogen receptor and progesterone receptor, AR expression is predominantly localized to the stromal cells [21, 22]. AR expression in the endometrium decreases steadily from the early proliferative phase to the mid-secretory phase [21]. Serum androgen levels change throughout the menstrual cycle, with levels peaking during ovulation [23, 24]. However, tissue dehydroepiandrosterone, androstenediol, androstenedione, and testosterone concentrations increase approximately by fourfold in secretory without significantly changing the plasma level [25]. Taken together, androgens could directly affect human endometrial functions.

Decidualization is the morphological and biological differentiation transformation of human endometrial stromal cells (HESCs) into specialized secretory cells during the secretory phase of the menstrual cycle; it is further characterized by the influx of specialized immune cells (uterine natural killer cells and macrophages) into the stroma, and vascular remodeling [26]. This process helps form a functional feto-maternal interface by controlling endovascular trophoblast invasion and tissue homeostasis and conferring resistance to environmental stress signals, including protection against oxidative cell death [27]. If impaired, several reproductive disorders, including implantation failure and recurrent miscarriage, can occur.

**Fig. 1:**
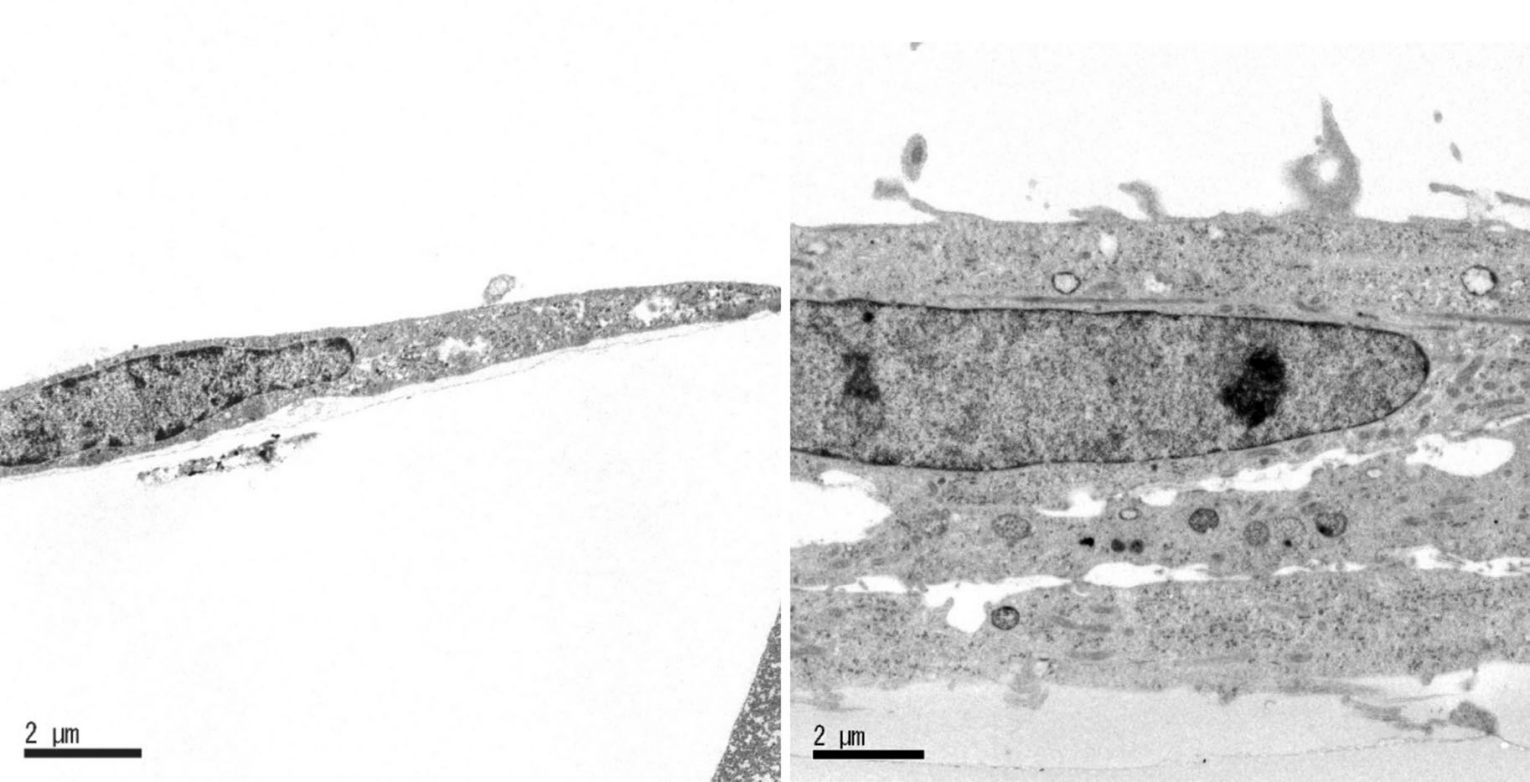
Ultrastructural appearance of confluent monolayers of human endometrial stromal cells (HESCs) treated with **A** vehicle control, **B** 8-bromoadenosine 3′,5′-cyclic monophosphate (8-br-cAMP) (0.5 mM), progesterone (P_4_) (10^−6^ M) and 5α-dihydrotestosterone (DHT) (10^−7^ M). The cells increased in size, forming two or three cell layers. The cytoplasm contained moderate amounts of rER, Golgi complexes, mitochondria, lysosomes, and others

**Fig. 2:**
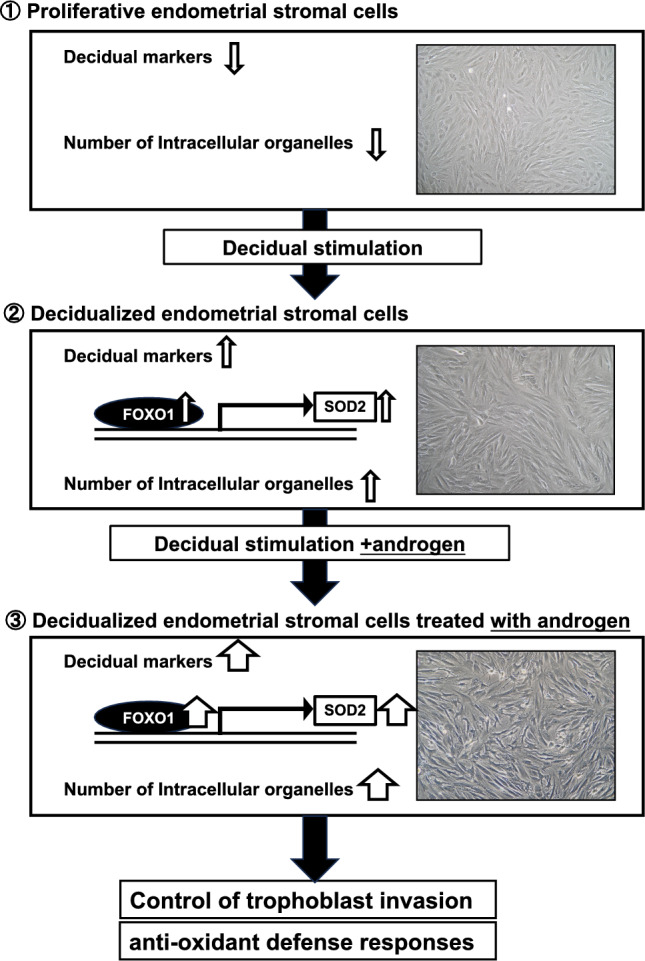
The effect of androgen on decidual phenotype of human endometrial stromal cells (HESCs) in vitro. Treatment of HESCs with or without 8-br-cAMP, P_4_ or in combination with a DHT. This transformation in vivo underpins the acquisition of specialized morphological characterizations and biological function

As mentioned above, AR expression is confined to the endometrial stromal compartment and is most pronounced during the proliferative phase. After ovulation, the AR decreases during the secretory phase [21, 22, 28], although its expression remains in the decidua of early pregnancy. Additionally, the role of AR signaling in early pregnancy is brought about by the observation that treatment of rats with anti-androgens delays implantation initiation, fetal development, and parturition. Furthermore, anti-androgens inhibit decidualization in pseudo-pregnant rats [29]. Although the AR decreases, the HESCs become increasingly responsive to androgens as they differentiate into decidual cells. The AR in decidual HESCs also regulates a relatively small but distinct group of genes involved in cytoskeletal organization, cell motility, and cell cycle progression [30]. Androgens were observed to enhance the expression of decidualization markers such as prolactin, IGFBP1, and FOXO1 and promote morphological and ultrastructural changes associated with the decidual process (e.g., expanded endoplasmic reticulum and increased numbers of mitochondria and lipid droplets) (Fig. 1) [31, 32]. Furthermore, androgens significantly decreased H_2_O_2_-induced apoptosis in decidualized HESCs dose-dependently accompanied with the increased expression of superoxide dismutase 2 (SOD2), which protects against oxidative stress [31]. Interestingly, DHT reportedly has no significant effect on trophoblast invasion in a co-culture system using the spheroid of HTR-8/Svneo trophoblast cells and decidualized HESCs. However, if HESCs were first decidualized in the presence of androgen, spheroid expansion was further stimulated [33]. Therefore, androgens may regulate the expansion and invasion of trophoblast cells through the decidual phenotype of HESCs. Taken together, androgens might play an important role in the menstrual cycle and the decidual process of endometrium (Fig. 2). However, additional investigations are needed to elucidate the precise role of androgens in endometrial decidualization.

## Androgens in the embryo and endometrium during implantation

Successful human implantation depends on the interaction between a developed embryo and a “receptive” endometrium. Endometrial receptivity occurs during a limited period in the cycle, considered as the “implantation window,” wherein the endometrium allows the blastocyst to be implanted. The endometrium becomes receptive approximately 6 days after ovulation and remains so for up to 2–4 days [34]. The implantation process is generally divided into three steps: apposition (the blastocyst is oriented correctly), adhesion (the blastocyst comes into contact with the epithelium and attaches to the endometrial surface), and invasion (the blastocyst penetrates the epithelium, invading the endometrial stroma) [35]. Aside from ovarian estrogen and progesterone, endometrial autocrine or paracrine factors and embryo-derived signals are also important for this process.

Polycystic ovaries syndrome (PCOS) affects approximately 5% of all women of reproductive age [36]. Its main clinical characteristics include hyperandrogenemia, oligo- or anovulation, infertility, enlarged polycystic ovary, hirsutism, obesity, and insulin resistance. Although the endocrine and clinical presentation of PCOS can be heterogeneous, hyperandrogenemia is the most responsible biochemical abnormality. Although ovulation occurs in approximately 80% of patients treated for PCOS, the pregnancy rate is only 40–50% [37]. Additionally, the miscarriage rate is reportedly 30–50% of all conceptions [38, 39]. The expression level of AR in the endometrium of patients with PCOS was elevated in comparison with that of fertile controls [40]. Furthermore, the putative biomarkers of endometrial receptivity, including αvβ3 integrin, glycodelin, estrogen receptor α, and HOXA10, are aberrantly expressed in patients with PCOS [41–43]. Thus, a disruption of endometrial development and receptivity may link to the decreased fertility and poor reproductive outcome in these patients. Moreover, high plasma concentrations of androgens are associated with adverse reproductive outcomes, including infertility and increased incidence of pregnancy loss with or without PCOS [44, 45]. Intriguingly, a recent cohort study based on Swedish nationwide register data showed that early initiation of anti-androgen treatment increased the chance of childbirth in patients with PCOS after spontaneous conception [46]. In pregnant rats, treatment with DHT (a more potent metabolite of testosterone) induced deficiencies in endometrial receptivity and mitochondrial function [47]. Furthermore, the expression of Wilms tumor suppressor (WT1), which is expressed during the window of implantation, in the endometrium was downregulated by androgens [48]. All of these observations point toward an adverse effect of androgens on endometrial function, which, in turn, may account for the association between hyperandrogenemia and subfertility or recurrent miscarriage. However, the precise mechanisms for hyperandrogenism and implantation remain unclear.

## Androgens and endometriosis

Endometriosis is one of the most prevalent gynecological disorders, affecting approximately 10% of women of reproductive age and with a prevalence rate of as high as 35–50% in women with endometriosis-associated infertility and/or pain [49]. The etiology of endometriosis has been explained by several theories. Of these theories, the most common and acceptable is the retrograde reflux of menstrual blood containing endometrial tissue via the fallopian tubes into the peritoneal cavity where it attaches to the peritoneum, proliferates, differentiates, and eventually invades the underlying tissue [50]. Although 90% of women of reproductive age have retrograde menstruation, only approximately 10% is diagnosed with endometriosis [51]. Retrograde menstrual flow is common, but it does not explain why only some women develop endometriosis. Therefore, other pathologic factors are required to establish this disease. The eutopic endometrium of women with endometriosis is believed to be abnormal, predisposing them to ectopic diseases. The phenotype for the differentiation capacity of ectopic endometrium is significantly varied in comparison with that of eutopic endometrium [52]. However, the pathogenesis of endometriosis has not been fully characterized.

The concentration of testosterone in endometriosis lesions was strikingly higher than that in the corresponding serum concentrations and eutopic endometrium of healthy controls [53]. Carnerio et al. demonstrated that AR and 5α-reductase, which is an enzyme essential for converting testosterone into the more potent androgen DHT, are localized in the cytoplasm of glandular and stromal cells of the ectopic endometrium [54]. Thus, active androgens may be formed in endometriosis tissue, and both local and systemic androgens may contribute to establishing and developing endometriosis. Additionally, a bioinformatic analysis identified AR as a key endometriosis-associated transcription factor, with 373 target AR genes significantly differentially expressed in endometriotic lesions compared with those in the normal endometrium [55]. Interestingly, polymorphic CAG repeats of AR genes may be related to the pathogenesis of endometriosis [56–58].

## Conclusions

This review highlights the studies demonstrating that androgens and their receptors play a crucial role in regulating the endometrial physiological and pathological conditions. Androgens enhance the decidual process in the endometrium. Conversely, several experiments point toward an adverse effect of androgens on implantation, which, in turn, may account for the association between hyperandrogenemia and subfertility or recurrent miscarriage. Therefore, the exact role of androgens on endometrial receptivity remains unclear. Additional studies are needed to confirm whether the physiological and supra-physiological concentrations of androgen have beneficial or adverse effects on endometrial receptivity. Meanwhile, our review also discusses the findings of studies on androgens in gynecological pathological conditions such endometriosis. Currently, the role of androgens in physiological and pathological endometrial functions is still controversial, requiring further clarification. Extensive basic and clinical research studies are required to elucidate the expression, regulation, and functions of androgens under normal and disease conditions to identify new biomarkers and robust therapeutic applications of androgens.
